# VEGF/Neuropilin Signaling in Cancer Stem Cells

**DOI:** 10.3390/ijms20030490

**Published:** 2019-01-23

**Authors:** Arthur M. Mercurio

**Affiliations:** Department of Molecular, Cell and Cancer Biology, University of Massachusetts Medical School, 364 Plantation Street, Worcester, MA 01605, USA; arthur.mercurio@umassmed.edu; Tel.: +01-508-471-6212

**Keywords:** neuropilin, VEGF, cancer stem cell

## Abstract

The function of vascular endothelial growth factor (VEGF) in cancer extends beyond angiogenesis and vascular permeability. Specifically, VEGF-mediated signaling occurs in tumor cells and this signaling contributes to key aspects of tumorigenesis including the self-renewal and survival of cancer stem cells (CSCs). In addition to VEGF receptor tyrosine kinases, the neuropilins (NRPs) are critical for mediating the effects of VEGF on CSCs, primarily because of their ability to impact the function of growth factor receptors and integrins. VEGF/NRP signaling can regulate the expression and function of key molecules that have been implicated in CSC function including Rho family guanosine triphosphatases (GTPases) and transcription factors. The VEGF/NRP signaling axis is a prime target for therapy because it can confer resistance to standard chemotherapy, which is ineffective against most CSCs. Indeed, several studies have shown that targeting either NRP1 or NRP2 can inhibit tumor initiation and decrease resistance to other therapies.

## 1. Introduction

Despite advances in the diagnosis, prognosis, and treatment of solid tumors, multiple issues continue to drive morbidity and mortality. Key challenges include resistance to therapeutic strategies, tumor recurrence and metastasis. These concerns are complicated by tumor heterogeneity. Tumor cells within a given tumor differ in morphology, proliferative capacity, sensitivity to therapeutic agents and metastatic potential [1]. One of the most telling aspects of tumor heterogeneity is the observation that only a subset of cells within a tumor is capable of initiating a new primary tumor, recurrence or metastasis [2,3]. This ability to initiate tumors de novo has led to the cancer stem cell (CSC) hypothesis. The consensus definition of a CSC is “a cell within a tumor that possesses the capacity to self-renew and to cause the heterogeneous lineages of cancer cells that comprise the tumor” [4]. CSCs can be defined experimentally by their ability to initiate a new tumor when transplanted as xenografts. For this reason, CSCs are often termed tumor initiating cells (TICs) or tumorigenic cancer cells. CSCs are resistant to standard chemo- and radiation therapy and are hence likely responsible for tumor recurrence [5], and they contribute to metastasis [6]. One compelling demonstration of the existence and importance of CSCs in breast cancer derives from the study of highly aggressive tumors such as triple negative breast cancers (TNBCs). TNBCs harbor a higher frequency of CSCs than other breast cancer subtypes, and this sub-population is likely responsible for their aggressive nature [7]. A critical issue that emerges from these observations is the nature of the mechanisms that sustain the function of CSCs. This issue is significant because the CSC state is plastic and it can be acquired or lost by perturbations in the tumor microenvironment [8]. Although multiple cell autonomous and non-cell autonomous mechanisms likely exist for sustaining CSCs, our efforts have been focused on the role of vascular endothelial growth factor (VEGF) signaling mediated primarily by the neuropilins (NRPs). In this review, I will summarize the knowledge that exists about this important signaling axis, discuss its potential impact on improving the clinical management of aggressive cancers and highlight key areas for future study.

## 2. VEGF

VEGF was characterized initially as an endothelial-specific mitogen that has the capacity to induce physiological and pathological angiogenesis, as well as vascular permeability [9,10]. This VEGF is now known as VEGF-A and is a member of a larger family of growth factors that also includes VEGF-B, VEGF-C, VEGF-D, and placental growth factor (PlGF). These family members differ in their expression pattern, receptor specificity, and biological functions [11]. VEGFA, often referred to as simply VEGF, has been studied the most intensely and exists as several distinct variants (VEGF_121_, VEGF_145,_ VEGF_148,_ VEGF_165_, VEGF_183_, VEGF_189_, and VEGF_206_) that arise from alternative splicing and also differ in receptor specificity and function [11]. Not surprisingly, the role of VEGFs in vascular and lymphangiogenesis has dominated the VEGF field since their initial discovery and these studies have yielded significant insight into the mechanisms that underlie the complex process of angiogenesis [12]. Importantly, this work provided the foundation for the development of anti-angiogenic therapies that target VEGF and VEGF receptors. It has become apparent, however, that the function of VEGF is not limited to angiogenesis and vascular permeability. VEGF, for example, can impact the function of immune cells present in the tumor microenvironment and, consequently, the host response to tumors (e.g., [13]) and VEGF receptors may regulate the function of fibroblasts in tumor stroma [14]. As alluded to above, one of the most exciting developments is the discovery that autocrine and paracrine VEGF signaling occurs in tumor cells and that this signaling contributes to key aspects of tumorigenesis, especially the function of CSCs, independently of angiogenesis [15]. This finding highlighted the importance of VEGF receptors on tumor cells in the context of their role in sustaining CSC function.

## 3. VEGF Receptors (VEGFRs and Neuropilins)

The classical VEGF receptors are the receptor tyrosine kinases (RTKs) VEGFR1 (Flt-1), VEGFR2 (Flk-1/KDR), and VEGFR3 (Flt-4) [16]. Although the expression of these receptors was initially thought to be limited to endothelial cells, it is now known that most of these receptors are expressed by many tumor types and that their expression correlates with clinical parameters [15]. VEGFR2 is the predominant RTK that mediates VEGF signaling in endothelial cells and drives VEGF-mediated angiogenesis [16]. Interestingly, some tumor cells express VEGFR2 and it can mediate VEGF signaling (e.g., [17,18]). There is also evidence that tumor cells can respond to autocrine and paracrine VEGF signals independently of the VEGFRs, which implies that other types of receptors mediate or contribute to VEGF signaling in these cells. In this context, the NRPs have garnered the most attention in recent years as VEGF receptors that function in tumor initiation and progression [19,20,21]. Interestingly, some of these studies, but not all, have highlighted a key role for the NRPs (NRP1 and NRP2) in mediating this signaling and discounted the contribution of VEGFRs, e.g., [22,23]. This intriguing observation underscores the emphasis of this review on VEGF/NRP signaling in CSCs.

There are two NRPs expressed in vertebrates (NRP1 and NRP2). These transmembrane glycoproteins that exhibit 44% identity at the amino acid level and they contain four distinct extracellular domains that mediate ligand binding and a short cytoplasmic domain that lacks known catalytic activity [24,25,26,27]. Alternative splicing of NRP1 and NRP2 can produce multiple isoforms including secreted, soluble forms and NRP2 variants with differences in their cytoplasmic domains [26]. As their name implies, the NRPs were characterized originally for their role in the developing nervous system. More specifically, they function as receptors for a class of axon guidance factors termed the semaphorins [28,29]. They appear to lack intrinsic signaling capacity and function primarily as co-receptors that impact the function of signaling receptors. For example, their ability to function as semaphorin receptors requires their association with specific plexins, which are transmembrane receptors that contribute to neuronal development by regulating guanosine triphosphatases (GTPases) [30,31].

The critical finding in the context of this review is that NRPs can function as VEGF receptors and that they are expressed on tumor cells [19]. This seminal finding launched studies aimed at understanding their contribution to tumor biology. The NRPs form complexes with VEGF RTKs (VEGFR1 and VEGFR2) and enhance their affinity for VEGF [32]. As will be discussed, the NRPs also can impact the function of many other receptors that are critical for tumor cell function and there is evidence that they may signal independently of other receptors. 

**Figure 1:**
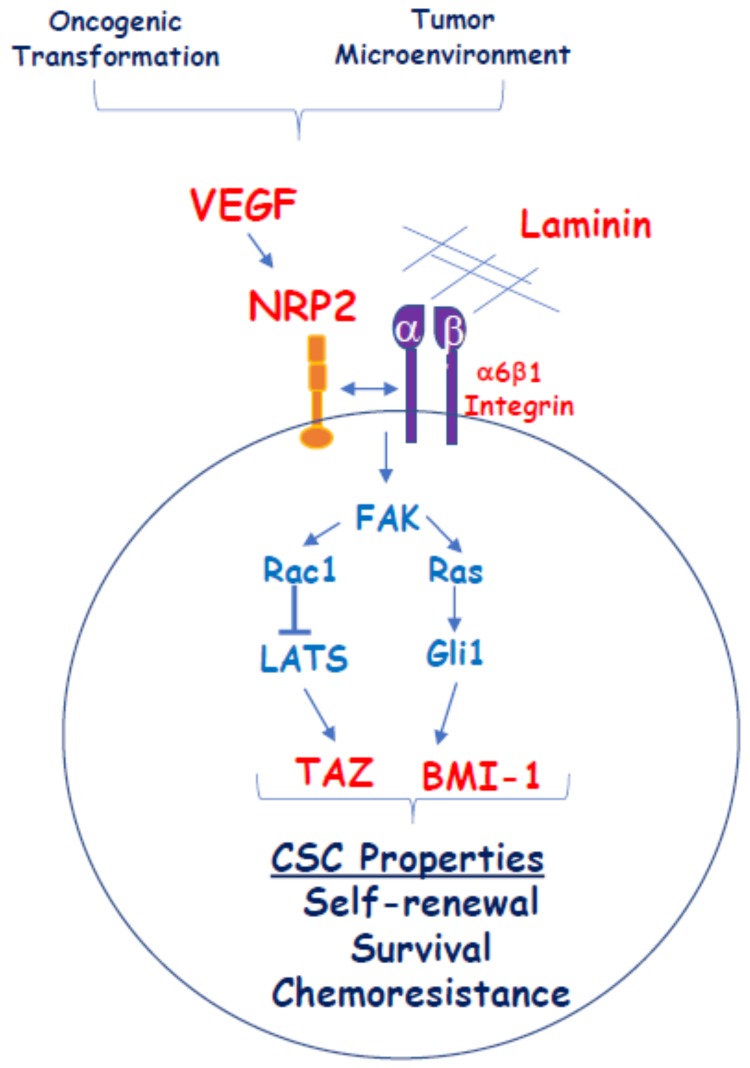
One proposed mechanism for how vascular endothelial growth factor/neuropilin (VEGF/NRP) signaling impacts cancer stem cell (CSC) function independently of VEGFRs. Oncogenic transformation and conditions in the tumor microenvironment result in the establishment of autocrine VEGF/NRP2 signaling in tumor cells that contributes to the genesis of a CSC phenotype. VEGF/NRP2 signaling facilitates α6β1 integrin engagement with laminins such as laminin 511 in a CSC niche. Signaling through this integrin activates a focal adhesion kinase/Ras pathway that promotes the Gli1-mediated expression of B lymphoma Mo-MLV insertion region 1 homolog (BMI-1), a stem cell factor. VEGF/NRP2 signaling also promotes the repression of LATS by Rac1 and the consequent activation of the stem cell factor and Hippo effector TAZ. The convergence of these signaling pathways contributes to the acquisition of stem cell properties including self-renewal, survival, and chemoresistance.

The fact that the expression of NRP1 and NRP2 is absent or low in many epithelial tissues but increased in carcinomas that originate from these tissues provides correlative support for their involvement in oncogenic processes [15]. Moreover, NRP expression is associated with more aggressive tumors and several studies have observed their preferential expression in CSCs. In breast cancer, for example, NRP2 is expressed preferentially in TNBCs compared to other breast cancer sub-types and it is enriched in breast CSCs [33]. A similar expression pattern has been observed in prostate cancer where NRP2 expression correlates with Gleason grade and it is also enriched in prostate CSCs [34]. These observations infer that specific mechanisms associated with oncogenic transformation and the genesis of CSCs induce NRP expression. Indeed, several studies have provided insight into these mechanisms. Notably, hedgehog (Hh) signaling can induce NRP expression [33,35], which may be part of a positive feedback loop because VEGF/NRP signaling can also induce expression of the Hh target gene Gli1 [33,35,36]. PTEN loss induces *NRP2* transcription in prostate cancer by a mechanism that involves the Jun N-terminal kinase (JNK)/c-jun pathway, providing a direct link between the loss of a tumor suppressor and induction of *NRP2* transcription [34]. Interestingly, both c-jun and Gli1 can bind the *NRP2* promoter and may function in concert to regulate *NRP2* transcription [33,34]. Expression of COUP transcription factor II (COUP-TFII) correlates with disease recurrence and progression in prostate cancer and it can directly stimulate the transcription of *NRP2* [37,38]. COUP-TFII can also suppress Notch signaling [39], which is interesting because there are reports that some Notch ligands (DLL-4) can repress VEGFR2 and NRP1 expression [40], but other ligands (DLL-1) can stimulate their expression [41]. Other studies have implicated Sox2 in regulating NRP1 expression in squamous carcinoma [42] and there is evidence for the regulation of NRP1 in hepatic CSCs by specific microRNAs [43]. Collectively, the existing data indicate that the induction of NRP expression is intimately associated with oncogenesis and the genesis of CSCs (Figure 1). 

## 4. Contribution of VEGF/NRP Signaling to CSCs

Although earlier studies had recognized the importance of VEGF/NRP signaling to the behavior of aggressive tumor cells and tumor development [15], the involvement of this signaling pathway in sustaining CSC function specifically was first realized in 2011 [44]. This study, which involved a rigorous analysis of the early stages of squamous carcinoma formation in the skin, resulted in several key seminal findings that implicated autocrine VEGF signaling in the function of cancer stem cells directly. In early stage tumors or papillomas, cancer stem cells are localized in a perivascular niche adjacent to endothelial cells. Blocking VEGFR2 reduced the size of the cancer stem cell pool and their self-renewal potential. Conditional deletion of *VEGFA* in tumor cells of established tumors caused regression by reducing both microvascular density and the proliferation and renewal of CSCs. Moreover, genetic deletion of *NRP1* prevented the ability of VEGF to promote stemness and self-renewal. A subsequent study described the importance of VEGF/VEGFR2/NRP1 signaling in the survival of glioma CSCs and tumor growth [18]. These findings established the importance of NRP1 in the context of VEGF-A signaling in CSCs. Another study implicated PlGF, a VEGF family member, and PlGF/NRP1 signaling in the aggressive behavior of medulloblastoma [22]. Although this study did not investigate CSCs directly, high NRP1 expression was shown to characterize the most aggressive tumors with poor overall survival. A later study, however, demonstrated the association of NRP1 with medulloblastoma CSCs and its involvement in their self-renewal [45]. NRP2 also contributes to CSC function. NRP2 is expressed preferentially on breast CSCs and VEGF/NRP2 signaling was shown to be important for the genesis of TNBCs and tumor initiation [33]. Another breast cancer study highlighted the role of VEGF-C/NRP2 signaling in enhancing CSC characteristics [46].

One outstanding issue with respect to VEGF/NRP signaling in CSCs is that the expression of both VEGF and the NRPs is not limited to CSCs in many tumors. In TNBC, for example, a high frequency of tumor cells express both VEGF and NRP2 but only a fraction of these cells has CSC properties [33]. This observation implies that qualitative or quantitative differences in VEGF/NRP signaling exist in CSCs compared to non-CSCs. Clearly, this issue merits further investigation. Another important issue is the relationship of VEGF/NRP signaling to the epithelial to mesenchymal transition (EMT) in the context of CSCs. Several studies have implicated the EMT in the genesis of CSCs, although this relationship may be more nuanced than originally thought [47]. Autocrine VEGF/NRP signaling can promote an EMT phenotype [48] and these two phenomena may function in concert to promote CSC properties [15].

Although not the focus of this review, a discussion of VEGF signaling in tumor cells must include mention of semaphorins, especially class III semaphorins (SEMA3s), because they are secreted by tumor cells, function as NRP ligands and have been implicated in tumor-associated functions (e.g., [49]). 

## 5. Mechanisms of VEGF/NRP Signaling in CSCs

Taken together, the studies described above affirm the importance of VEGF/NRP signaling in the function of CSCs and tumor development. Given that NRPs lack known intrinsic signaling capacity and function primarily as co-receptors, an important issue is how NRPs signal in CSCs. As mentioned, there is evidence that NRP1 can function in concert with VEGFR2 to affect CSC function, but there is also compelling evidence that VEGF/NRP signaling in CSCs can occur independently of VEGFRs. In this context, there is evidence that NRPs can influence the signaling properties of specific integrins that contribute to the function of CSCs. For example, NRP2 can interact with and function as a co-receptor for the α6β1 integrin in breast cancer cells [50]. This interaction facilitates α6β1 signaling, including its ability to activate focal adhesion kinase (FAK) [33,50] (Figure 1). These observations are relevant because NRP2 and the α6β1 integrin are markers of breast CSCs [33,51]. A subsequent study verified the role of NRPs in regulating α6 integrin signaling in epidermal CSCs. Interestingly, this study also concluded VEGF-NRP signaling activates FAK and that this signaling does not involve VEGFR [52]. One major difference between this study and the previous report on the α6β1 integrin, however, is that this study argued that the α6β4 integrin interacts with NRP1 on the surface of CSCs. This discrepancy may be the result of differences in α6 integrin expression between breast and epidermal CSCs. Nonetheless, these studies demonstrate that NRPs can promote VEGF signaling without VEGFR involvement by co-opting integrin signaling (Figure 1). There is also evidence that NRP1 can facilitate EGFR signaling in cancer cells [53], although this mechanism has yet to be demonstrated in CSCs. Another aspect of NRP1 and NRP2 signaling is that these receptors can regulate the trafficking and, consequently, intracellular signaling of growth factor receptors including VGFR2 [18] and EGFR [54].

The possibility exists that NRPs signal independently. Specifically, the cytoplasmic domains of NRP1 and NRP2 contain a PDZ binding domain that can bind PDZ-containing proteins, especially neuropilin interacting protein (NIP; also known as GIPC1). GIPC1 is a cytoplasmic scaffolding protein that interacts with a broad range of receptors and contributes to receptor trafficking and signal transduction [55,56], and it has been implicated in tumorigenesis [56]. One study concluded that the ability of NRP1 to mediate PlGF-stimulated growth of medulloblastoma requires its PDZ binding domain and is independent of VEGFR1 activity [22]. Presumably, this motif functions by forming scaffolding complexes that transduce NRP signals, a possibility supported by the finding that GIPC1 mediates the interaction of NRP1 with c-abl, a tyrosine kinase that could mediate NRP1 signaling [14]. GIPC1 can also function as a ‘bridge’ to promote the association of receptors that contain PDZ-binding domains such as NRPs and integrins [52,57]. This latter scenario may be relevant to the association of NRP2 with the α6β1 integrin because the α6 cytoplasmic domain contains a PDZ-binding domain [58]. Interestingly, the NRP2 cytoplasmic domain contains a motif with partial consensus to an immunoreceptor tyrosine-based activation motif (ITAM) that can mediate signaling in other receptors [59], although there is no evidence yet that it is functional. 

## 6. Effector Mechanisms

How VEGF/NRP signaling impacts the self-renewal and survival of CSCs is a problem of paramount importance that has been addressed in relatively few studies. One example of such a mechanism derives from work on VEGF/NRP2 activation of the α6β1 integrin. This activation induces a FAK/Ras signaling pathway that culminates in non-canonical hedgehog (Hh) signaling that activates Gli1, which induces expression of Bmi-1 [33], a Polycomb group transcriptional repressor that has been implicated in self-renewal and tumor initiation [60,61]. Gli1 can stimulate the transcription of *NRP2* [33,35], creating a positive feedback loop that has the potential to sustain the self-renewal function of CSCs. Hh signaling also has a critical role in tumor-stromal interactions in this context as evidenced by the finding that tumor-derived sonic Hh stimulates PlGF expression in stromal cells, which promotes the growth of medulloblastomas [22]. More recently, it was reported that autocrine VEGF signaling mediated by NRP2 promotes the self-renewal of breast CSCs by sustaining activation of the hippo pathway transducer TAZ [23]. This finding is significant because TAZ has been shown to be necessary for the genesis of breast and other CSCs [62,63]. Importantly, it also links a key component of the tumor microenvironment (VEGF) with TAZ activation as a mechanism that underlies CSC function. This study highlighted a critical role of the Rac1 GTPase in TAZ activation by VEGF-NRP2 signaling, which is consistent with the key role for Rho family GTPases in promoting YAP/TAZ activation observed in endothelial cells [21]. An essential component of this mechanism is the repression of β2-chimaerin, a Rac GAP, by TAZ and the consequent activation of Rac1 resulting in a positive feedback loop driven by VEGF-NRP2 signaling that sustains TAZ activation [23]. 

An interesting connection between VEGF-C/NRP2 signaling and oxidative stress in breast CSCs has also been reported [46]. Specifically, this signaling axis regulates the expression of superoxide dismutase 3 (Sod3), which promotes resistance to oxidative stress and, consequently, enhances the survival of CSCs. There is also suggestive evidence that VEGF/NRP1 signaling confers CSC properties in breast cancer by activating Wnt/β-catenin signaling [64].

Although the existing data provide some insight into the effector mechanisms that enable VEGF/NRP signaling to promote CSC properties, much remains to be learned and several key questions need to be addressed. More work is needed to determine if the known mechanisms and mechanisms to be determined can be integrated into a broad network. Clearly, however, these mechanisms may differ depending on tumor type and the nature of NRP signaling employed by specific tumor cell populations. Also, NRP1 and NRP2 likely differ in their ability to promote CSC function and this difference may reflect differences in their expression patterns. It is also possible that they differ in their signaling properties. If so, what is the structural basis for this difference given their high degree of homology. Another issue is how the different VEGF variants differ in their ability to affect CSC function. NRP1 and NRP2 can bind multiple spice variants of VEGF-A, as well as other VEGF family members including VEGF-C and PlGF [27]. Indeed, there is evidence that VEGF-A [33], VEGF-C [46], and PlGF [22] signaling can contribute to CSC function but comparative studies have not been done. As mentioned, splice variants of the NRPs exist and these may differ in function. For example, there is evidence that the NRP2b splice variant contributes to properties associated with a CSC phenotype in lung cancer (therapy resistance, mesenchymal phenotype, and poor survival), properties that cannot be mediated by NRP2a [65]. Interestingly, NRP2b is unable to interact with GIPC, which highlights the need for a better understanding of how NRPs signal in different cellular contexts.

Another area that has received scant attention is the contribution of the tumor microenvironment to VEGF/NRP signaling and the regulation of CSC function. The notion of a CSC ‘niche’ comprised of stromal cells and extracellular matrix proteins implies that this niche sustains CSC function [66]. There is evidence, for example, that the matrix protein laminin 511 is a component of a CSC niche and that the engagement of this laminin with a splice variant of the α6β1 integrin (α6Bβ1) sustains TAZ activity in breast CSCs, most likely in concert with NRP2 [23,67]. The hypoxic microenvironment that exists in many tumors has been shown to promote and sustain CSC function [68]. One mechanism may involve its effect on VEGF/NRP signaling. Hypoxia-inducible factor (HIF)-mediated transcription is a major driver of VEGF expression in tumors [69], and it is likely that hypoxia contributes to the establishment of autocrine and paracrine signaling networks in tumor cells.

## 7. Chemoresistance and Therapy

One of the defining characteristics of CSCs is their resistance to standard chemo- and radiation therapy [5]. For this reason, there is considerable interest in elucidating the mechanisms that contribute to this resistance and targeting these mechanisms for more effective clinical management, especially of aggressive tumors. Not surprisingly based on the information provided in this review, VEGF/NRP signaling has been shown to have a key role in therapy resistance and there is considerable interest in targeting this pathway in tumor cells [15,70]. Indeed, several studies have shown that targeting either NRP1 or NRP2 can inhibit tumor initiation and decrease resistance to other therapies [15,70]. The use of anti-VEGF therapy to inhibit CSC function, however, is hindered by the finding that the most common anti-VEGF drug (bevacizumab) blocks the binding of VEGF to receptor tyrosine kinases but not to NRPs [71]. This observation may explain, in part, the dismal efficacy of bevacizumab for many cancers [72,73], and it reinforces the potential benefit of targeting the NRPs directly as an approach to inhibiting VEGF signaling in CSCs. This point is exemplified by the finding that prostate cancer cells selected for their resistance to bevacizumab and sunitinib, a VEGFR inhibitor, are enriched for stem cell properties and NRP signaling [74]. More importantly, it was observed that NRP signaling induces expression of P-Rex1, a Rac1 guanidine exchange factor, and that Rac1-mediated ERK activation is responsible for resistance to bevacizumab and sunitinib. These findings revealed a role for VEGF/NRP-mediated regulation of P-Rex1 in the biology of CSCs and resistance to therapy. An intriguing aspect of this study is the ‘VEGF paradox’. Specifically, resistance to VEGF-targeted therapy (bevacizumab and sunitinib) is mediated by an enhancement of VEGF/NRP signaling. In fact, prostate cancer cells treated with bevacizumab and sunitinib exhibit a marked increase in VEGF expression despite the fact that bevacizumab targets the interaction of VEGF with VEGFRs [75]. One interpretation of these data is that neither bevacizumab nor sunitinib is effective at targeting prostate cancer cells with stem cell properties and that the CSC population, which is characterized by autocrine VEGF/NRP signaling, is enriched by treatment with these drugs because they target primarily non-CSCs. This study also provided evidence that bevacizumab or VEGFR-targeted therapy in prostate cancer is more efficacious if it is combined with targeted inhibition of the NRP2 effectors P-Rex1 and Rac1 [74].

A more recent study implicated NRP1 in adaptive resistance to oncogene-targeted therapies [76]. It was reported that treatment of addicted melanoma cells with BRAF inhibitors and breast cancer cells with anti-HER2 drugs resulted in increased NRP1 expression, which was shown to have a causal role in resistance to these therapies. Specifically, NRP1 stimulated a JNK-signaling pathway that resulted in the upregulation of alternative effector kinases (EGFR or IGF1R) that sustained tumor growth. Most importantly, combining ant-NRP1 therapy with oncogene-targeted therapies prevented the resistance to these therapies. Although this study did not investigate CSCs specifically, it seems likely based on other studies that the resistant cells had acquired stem cell properties coincident with NRP1 signaling.

## 8. Summary

VEGF/NRP signaling appears to be a defining characteristic of CSCs in multiple tumor types that is necessary to sustain their self-renewal and survival. Moreover, the acquisition of VEGF/NRP signaling in tumor cells is intimately associated with oncogenic transformation and conditions in the tumor microenvironment. There is also compelling evidence that VEGF/NRP signaling is a prime target for therapy, especially the use of reagents that inhibit NRP function. These reagents may be most efficacious when used in combination with drugs that target non-CSCs or oncogene-addicted pathways as a means to overcome therapy resistance.

Despite the progress that has been made in assessing the importance of VEGF/NRP signaling in CSCs, much remains to be learned. Key areas for future work include a better understanding of the nature of NRP signaling and how NRP1 and NRP2 signaling differ in this regard. One area in particular is a more rigorous investigation of how NRPs signal independently of VEGFRs and whether they possess intrinsic signaling capacity. Also, the mechanisms by which VEGF/NRP signaling impacts the self-renewal and survival of CSCs needs more study. In this direction, emphasis should be on the relationship of VEGF/NRP signaling to other mechanisms that have been implicated in CSC function.
